# The CAPRA-S score versus subtypes of minimal residual disease to predict biochemical failure after radical prostatectomy

**DOI:** 10.3332/ecancer.2020.1063

**Published:** 2020-06-25

**Authors:** Nigel P Murray, Socrates Aedo, Cynthia Fuentealba, Eduardo Reyes, Anibal Salazar, Eghon Guzman, Shenda Orrego

**Affiliations:** 1 Faculty of Medicine, University Finis Terrae, Providencia, Santiago 7501015, Chile; 2 Urology Service, Hospital DIPRECA, Las Condes, Santiago 7770199, Chile; 3 Faculty of Medicine, University Diego Portales, Santiago 7770199, Chile; 4 Department of Urology, Hospital de Carabineros de Chile, Ñuñoa, Santiago 8370179, Chile; 5 Faculty of Medicine, University Mayor, Providencia, Santiago 7601003, Chile; a https://orcid.org/0000-0001-8154-8550; b https://orcid.org/0000-0003-4100-6997; c https://orcid.org/0000-0001-8430-3030; d https://orcid.org/0000-0001-9319-4219; e https://orcid.org/0000-0001-5012-6945; f https://orcid.org/0000-0003-2860-2954

**Keywords:** prostate cancer, biochemical failure, CAPRA-S score, minimal residual disease, circulating tumour cells, micrometastasis

## Abstract

**Objective:**

The objective of this study was to compare the CAPRA-S score (based on clinicopathological findings) and the subtypes of minimal residual disease (MRD) (based on the biological properties of cancer cells) to predict biochemical failure (BF) after prostatectomy radical.

**Patients and methods:**

This was a prospective single-centre study of men who underwent radical prostatectomy. One month after surgery, the blood and bone marrow were taken for circulating prostate cell (CPC) and micrometastasis detection, identified using anti-PSA immunocytochemistry and defined as positive or negative. Patients were classified as Group A: CPC and micrometastasis negative, Group B: micrometastasis positive and CPC negative and Group C: CPC positive. CAPRA-S scores were classified as low, intermediate and high risk. Kaplan–Meier curves for biochemical failure-free survival (BFFS) and restricted mean survival time (RMST) to biochemical failure were determined and compared for up to 10 years.

**Results:**

347 men participated with a median follow-up of 7 years, BFFS decreased proportionally with increasing CAPRA-S score and HR 1.13 and 1.65 for intermediate and high risk, respectively. After 10 years, the BFFS and RMST were 68%, 47% and 16% and 9, 7 and 6 years, respectively. The BFFS curves for MRD were not proportional; Group A and B BFFSs were similar up to 5 years, and then, there was an increasing failure in Group B patients After 10 years, the BFFS and RMST were 95%, 57% and 27% and 10, 9 and 6 years respectively. The CAPRA-S score failed to distinguish between Groups A and B, and one-third of high-risk Group C had low-risk CAPRA-S scores. MRD hazard ratios were Group B 1.76 and Group C 4.03.

**Conclusions:**

The MRD prognostic classification was superior to the CAPRA-S score in predicting BFFS and differentiated between early and late BF. The results need to be confirmed in larger studies.

## Introduction

Radical prostatectomy is a treatment option for clinically localised prostate cancer; however, approximately 20% of men will develop biochemical failure, defined as two or more consecutive prostate specific antigen (PSA) values of >0.20 ng/mL within 5 years of treatment [1, 2]. 25% of all biochemical recurrences occur between 5 and 10 years post-surgery [3, 4]. This is due to the dissemination of cancer cells before surgery. After a variable time period, these cancer cells proliferate resulting in an increase in serum PSA levels. This micrometastasis, which may be local, near to the prostate bed or distant such as in bone marrow, is termed as minimal residual disease (MRD). To individualise patient care, it is important to identify men at high risk of biochemical failure as early androgen blockade or radiotherapy has been reported to have a greater benefit [5, 6], as well as those at low risk of biochemical failure, who could be spared the potential side effects of therapy. The cancer of the prostate risk assessment score (CAPRA-S) incorporates the pathological findings found at surgery to predict the risk of biochemical recurrence and has been externally validated [7, 8]. It has been reported that there are two subtypes of MRD, one with circulating prostate cells (CPCs) and associated with early failure and a second type where only bone marrow micrometastasis is detected and is associated with late failure [9]. The detection of CPC post-prostatectomy when combined with the CAPRA-S score results in a significantly increased discriminative ability in establishing the probability of biochemical failure [10].

The objective of this present study was to establish and compare the predictive value of the CAPRA-S score and the sub-types of MRD to determine the risk of biochemical failure in men who had undergone radical prostatectomy as monotherapy for prostate cancer.

## Methods and patients

We conducted a prospective, observational single-centre study of men who underwent radical prostatectomy as the sole treatment for prostate cancer between 2000 and 2010. The study was approved by the local ethics committee and complied with the Declaration of Helsinki.

For each patient, after giving informed written consent, the following were recorded: date of radical prostatectomy, age and the clinicopathological details to calculate the CAPRA-S score as originally described [7]. a) serum total PSA (ng/mL) at the time of diagnosis (Advia CentaurXR® assay, Siemens Healthcare, Camberley, UK)

b) The pathological study of the surgical piece was performed by dedicated genitourinary pathologists according to the Gleason system 2005

i) presence or absence of extracapsular extension (ECE),

ii) presence or absence of positive surgical margins, defined as one with cancer cells in contact with the inked surface of the specimen.

iii) infiltration of the seminal vesicles and lymph nodes

## Detection of secondary circulating prostate cells

One month after radical prostatectomy, an 8 mL venous blood sample was collected in EDTA (Vacutainer®; Becton-Dickinson, Franklin Lakes, NJ, USA) and processed within 48 hours. CPC detection was independently evaluated with the evaluators being blinded to the clinical details.

### Collection of CPCs

Mononuclear cells were obtained by differential centrifugation using Histopaque 1,077 (Sigma-Aldrich, St Louis, MO, USA), washed and resuspended in a 100 μL aliquot of autologous plasma. 25 μL aliquots were used to make slides (silanised, DAKO, Carpinteria, CA, USA), were dried in air for 24 hours and fixed in a solution of 70% ethanol, 5% formaldehyde and 25% phosphate-buffered saline (PBS) pH 7.4 for 5 minutes and finally washed three times in PBS pH 7.4.

### Immunocytochemistry

**Figure 1.**
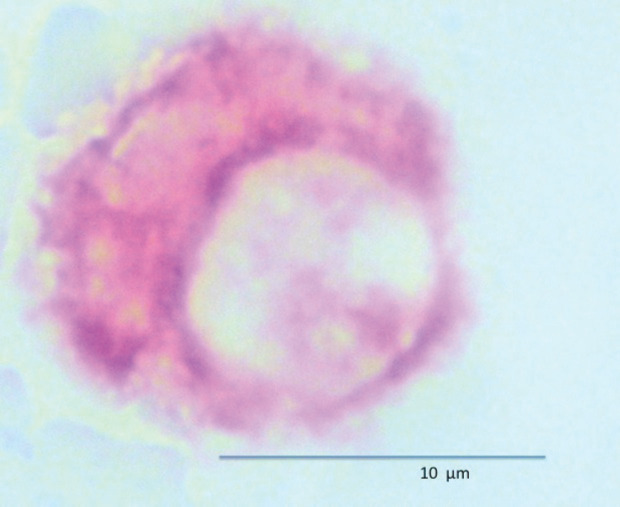
Circulating prostate cell expressing PSA (red) and negative for membrane CD45 (brown).

**Figure 2.**
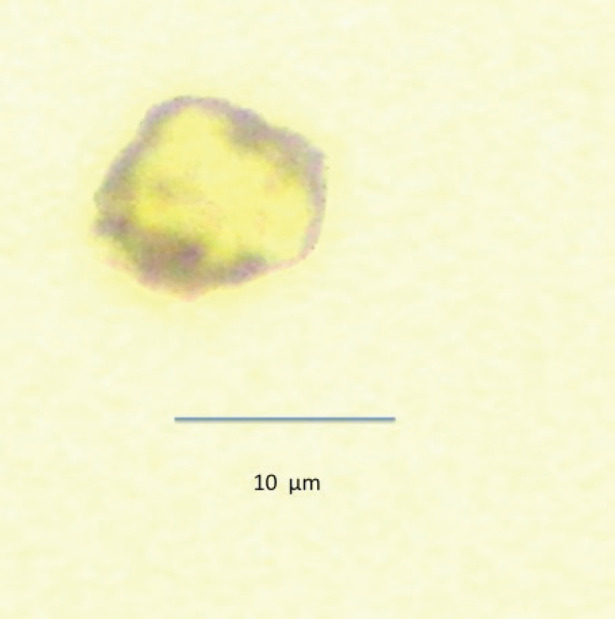
Leukocyte negative for PSA (red) and positive for membrane CD45 (brown).

CPCs were detected using a monoclonal antibody directed against PSA, clone 28A4 (Novocastro Laboratory, Newcastle, UK) and identified using an alkaline phosphatase-anti alkaline phosphatase-based system (LSAB2, DAKO, USA), with new fuchsin as the chromogen. The positive samples underwent a second process with anti-CD45 clone 2B11 + PD7/26 (DAKO, USA) and were identified with a peroxidase-based system (LSAB2, DAKO, USA) with DAB (3,3 diaminobenzidine tetrahydrochloride) as the chromogen. CPCs were defined according to the criteria of International Society of Hemotherapy and Genetic Engineering [11]. A CPC was defined as a cell that expressed PSA but not CD45; a leukocyte did not express PSA but expressed CD45 (Figures 1 and 2). A test was considered positive for CPCs when at least one cell/8 mL of blood was detected; the number of CPCs detected/8 mL blood sample was registered.

## Detection of bone marrow micrometastasis

**Figure 3.**
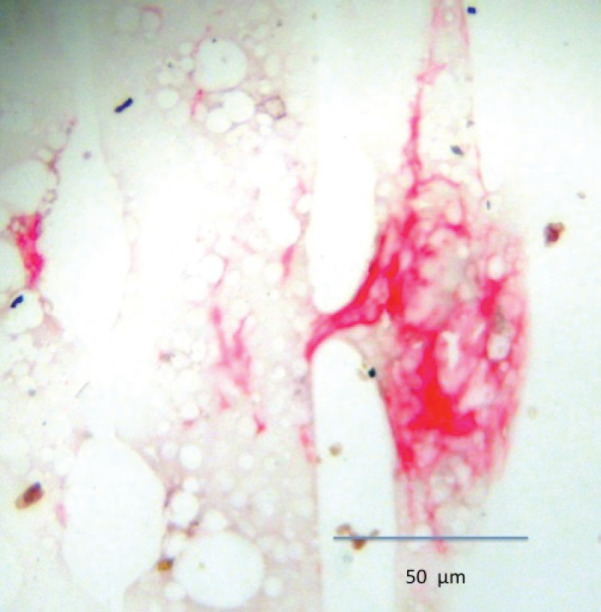
Bone marrow micrometastasis expressing PSA (red) and negative for membrane CD45 (brown).

**Figure 4.**
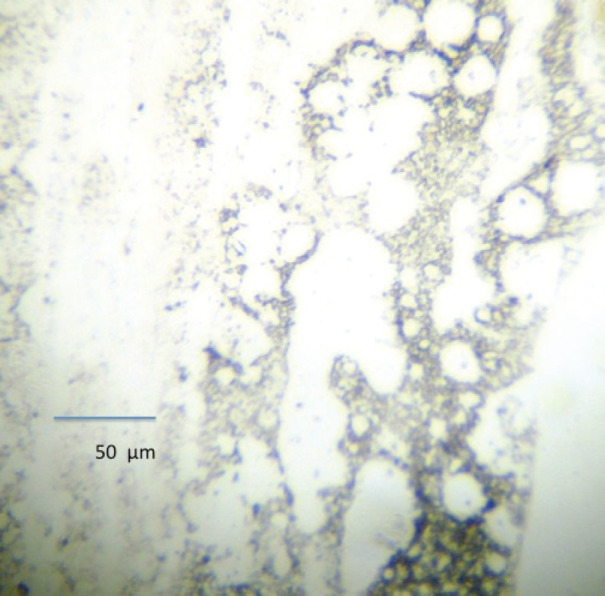
Bone marrow negative for micrometastasis.

Previous studies have used bone marrow aspirates to detect micrometastasis; however, prostate tumour cells detected in bone marrow aspirates are reported to be phenotypically different than those prostate cells detected in bone marrow biopsies and may not represent ‘true’ micrometastasis but rather cells circulating within the bone marrow [12]. For this reason, bone marrow biopsy ‘touch preps’ were used as the sample to test for micrometastasis. The biopsy was taken from the posterior superior iliac crest 1 month after surgery, and the sample was used to prepare four ‘touch preps’ using sialinised slides (DAKO, USA) and the four slides were processed as described for CPCs. A micrometastasis was defined as cells staining positive for PSA and negative for CD45 (Figures 3 and 4).

The slides for both CPCs and micrometastasis were analysed manually, the stained cells were photographed using a digital camera and from the images, it was determined if CPCs and/or micrometastasis were present or absent by one trained observer.

## Classification of patients

**a) CAPRA-S:** the subjects were divided into three CAPRA-S score groups: Group 1: CAPRA-S score between 0 and 2, Group 2: CAPRA-S score 3–5 and Group 3: CAPRA-S score 6–12.

**b) Minimum residual disease:** patients were divided into three MRD prognostic subgroups; Group A: negative for both CPCs and micrometastasis, Group B: CPCs negative but micrometastasis positive and Group C: CPCs positive with or without micrometastasis detected.

## Study endpoint

The primary study end-point was the presence of biochemical recurrence and secondary endpoint the mean time to failure after primary treatment.

## Statistical analysis

The analysis was performed using the program Stata/SE 16.0 for Windows (Stata Corp LLC). The quantitative and ordinal variables were described with respective central tendency and dispersion measurements [13], whereas nominal variables were described as proportions with their respective confidence intervals [13].

The prognostic groups were compared for age, total serum PSA, pathological Gleason score, pathological stage, ECE, surgical margins, seminal vesicle and lymph node infiltration. The Marascuillo procedure and Fishers’ exact tests were used for comparing multiple proportions. The Kruskal–Wallis test was used to test whether samples originate from the same distribution. A *p*-value <0.05 was taken to signify statistical significance, and all tests were two-tailed [13].

In the whole cohort and by MRD prognostic and CAPRA-S Score groups, a nonparametric biochemical failure-free survival analysis was performed at 10 years of follow-up, establishing the biochemical failure-free survival proportion of Kaplan–Meier and restricted mean survival time (RMST) [13, 14]. The RMST to 10 years establishes the expected time to the event during 10 years of observation, and its value is the area under the Kaplan–Meier nonparametric survival curve [14]. A non-parametric comparison (test Log-rank) of the biochemical failure-free survival by MRD prognostic and CAPRA-S score groups was performed. [13, 14]

Multivariable survival analyses are generally carried out using Cox regression. However, according to the proposed hypothesis with ‘dormant minimal residual disease’, there should be a period of time where the prognosis groups A and B should show a similar biochemical failure-free survival curve; at some time later, the biochemical failure-free survival curves separate with Group B patients showing a worse survival (CPCs negative micrometastasis positive). This situation breaches the assumption of proportional risks for using the Cox regression model [13, 14] and as such cannot be used.

An alternative to the Cox model, known as a flexible parametric survival model (FP model), permits the prediction (not descriptive like Kaplan–Meier model) of survival when there is no compliance with the proportional risk assumption [15–17]. The FP model is a regression method, in which the dependent variable is the survival for the studied outcome. This method uses the transformation of the independent variable (restricted cubic splines) and its iteration respective with time [15, 17]. Transformations of the independent variables generate different FP models. The degrees of freedom (DF) and the degrees of freedom for each time-dependent effect (DFTVC) indicate the transformations (number of knots) of the independent variables [15, 17].

For the prediction of biochemical failure, for a follow-up time of 10 years by MRD prognostic groups, a FP model was built using the following dummy independent variable: CPCs negative and micrometastasis positive (prognostic group B) and CPCs positive (prognostic group C). For the CAPRA-S score groups, a second FP model was built considering the following independent variables: CAPRA-S score 3–5 (CAPRA-S score Group 2) and CAPRA-S score 6–12 (CAPRA-S score Group 3)

The calibration aspect of the model refers to agreements between the predicted outcome and observed outcome [18]. We assess the calibration in the two FP predicted model by graphics comparing predicted FP survival model and observed Kaplan–Meier survival model

The discrimination of a prognostic model reflects its ability to distinguish between patient outcomes. We assessment the discrimination on the two FP predicted models using the Harrell’s C discrimination index [18],

From the FP predicted biochemical failure-free survival model for up to 10 years, the RMST and survival proportion were determined for each prognostic group of MRD and similarly for the CAPRA-S subgroups.

The decision curve analysis [19] is a method to evaluate and compare the prediction models and to determine the clinical consequences, i.e., treated or not treated. A decision curve analysis was performed for the two predictive models, comparing the clinical utility of survival of the prognostic groups and CAPRA-S groups.

## Results

A total of 538 subjects were recruited, of these 347 men underwent radical prostatectomy as monotherapy. These 347 men had a median follow-up of 7.53 years (IQR 4.81 years). The mean age was 65.5 ± 8.28 years with a median serum PSA of 5.51 mg/dL (IQR 3.26 ng/mL).

Two hundred and forty-five subjects (70.61%; 95%CI: 65.81 to 73.99) had a Gleason score of ≤6; pT2 tumours were present in 279 subjects (80.40%; 95% CI: 76.23–84.58). Extracapsular extension was present in 139/347 (40.06%, 95% CI: 34.90–45.21); positive surgical margins in 59/347 (17.00%, 95% CI: 13.05–20.96), seminal vesicle invasion in 10/347 (2.88%, 95% CI: 1.12–4.64) and lymph node infiltration in 4/347 (1.15%, 95% CI: 0.03–2.28).

CPCs were detected in 136 men (39.19%; 95% CI: 34.06– 44.33) and micrometastasis in 150 men (43.23%; 95% CI: 38.02–48.44). The MRD prognostic groups were, respectively, Group A: 155 subjects (44.67%; 95% CI: 39.44–49.90), Group B: 56 subjects (16.14%; 95% CI: 12.27–20.00) and Group C: 136 subjects (39.19%; 95% CI: 34.06 to 44.33).

The CAPRA-S score groups were, respectively, Group 1 (CAPRA-S score between 0 and 2): 234 subjects (67.44%; 95% CI: 62.50–72.37), Group 2 (CAPRA-S score between 3 and 5): 70 subjects (20.17%; 95% CI: 15.95–24.40) and Group 3 (CAPRA-S score between 6 and 12): 43 subjects (12.39%; 95% CI: 8.93–15.86).

Table 1 shows the comparison between the MRD prognostic groups. There were significant differences on the serum total PSA, Gleason score and CAPRA-S score between groups A versus C and B versus C. The presence of ECE and positive surgical margins showed significant differences between groups A versus C and groups B versus C. Seminal vesicle and lymph node infiltration were only present in group C with significant differences. The pathological stage showed a significant difference between groups: A versus B, A versus C and B versus C. There was no significant difference in the distribution of CAPRA-S scores between MRD Group A and B patients, both differing significantly from MRD Group C patients, where there was a significantly higher number of high-risk CAPRA-S scores. However, even in CPC-positive patients, over one-third were classified as low risk using the CAPRA-S score.

After 10 years of follow-up, the observed Kaplan–Meier biochemical failure-free survival and observed restricted mean biochemical failure-free survival time (area under the Kaplan–Meier nonparametric survival curve) according to MRD prognostic groups and CAPRA-S score groups are shown in Table 2.

The Log-rank test showed a *p*-value less than 0.01 comparing the biochemical failure-free survival between the MRD prognostic groups and the CAPRA-S score groups. There are significant differences between the two classification systems, in the CAPRA-S classification with increasing the risk score, the biochemical failure-free survival and restricted mean survival time decrease. This differs from the MRD classification, in which although with increasing risk group, the biochemical failure-free survival decreases, the restricted mean survival times for Group A and B are similar.

The flexible parametric (FP) survival model for the prediction of biochemical failure at 10 years by MRD prognostic groups showed two degrees of freedom for the restricted cubic spline function used for the baseline hazard rate (DF2). This incorporated the following coefficients: a) CPCs negative and micrometastasis positive (prognostic group B): hazard ratio of 1.76 (*p*-value < 0.01) and b) CPCs positive (prognostic group C): hazard ratio of 4.03 (*p*-value < 0.01).

The flexible parametric (FP) survival model for the prediction of biochemical failure at 10 years by CAPRA-S score groups showed one degree of freedom for the restricted cubic spline function used for the baseline hazard rate (DF1). This incorporated the following coefficients: a) CAPRA-S score between 3 and 5 (CAPRA-S score group 2): Hazard ratio 1.13 (*p*-value < 0.01) and b) CAPRA-S score between 6 and 12 (CAPRA-S score group 3): Hazard ratio 1.65 (*p*-value < 0.01).

**Figure 5.**
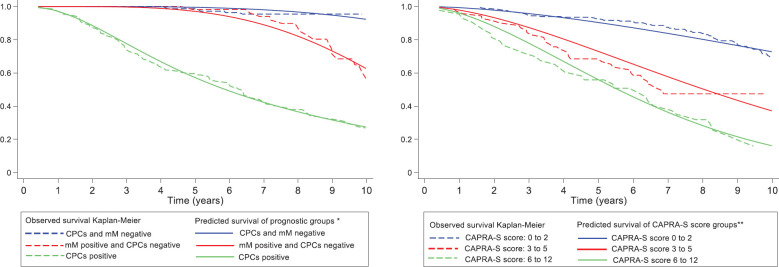
Observed biochemical failure-free survival curves (Kaplan–Meier) versus predicted biochemical failure-free survival curves for FP model by MRD prognostic groups and FP model by CAPRA-S score groups on 347 men with and without biochemical failure treated by radical prostatectomy for prostate cancer followed for 10 years.

There was agreement comparing the FP predictive model with the observed survival (Kaplan Meier) for MRD prognostic groups with a Harrell’s C index of 0.93 (considered very good). There was agreement comparing the predictive and observed survival for the CAPRA-S groups with a Harrell’s C index of 0.69 (considered acceptable). (Figure 5, Table 2). Table 2 shows the biochemical failure-free survival and mean restricted biochemical failure-free survival times for the differing MRD prognostic groups and CAPRA-S scores. Figure 5 shows the difference between the two classifications; with the CAPRA-S, the three curves are proportional with decreasing biochemical failure free survival and decreasing restricted mean biochemical failure-free survival times with increasing CAPRA-.S score. The curves for the MRD classification are significantly different, in which patients in Group B (CPC negative micro-metastasis positive) have a similar biochemical failure-free survival curve to those patients with MRD negative (Group A) for the first 5 years; thereafter, there is a divergent pattern with increasing biochemical failure in Group B patients.

MRD = minimal residual disease; FP = flexible parametric; CPCs = secondary circulating prostate cells; mM= micrometastasis; *Predicted FP model that incorporating: mM positive and CPCs negative (prognostic group B), CPCs positive (prognostic group C) with two degrees of freedom for the restricted cubic spline function used for the baseline hazard rate (DF2) and also, consider the CPCs positive (prognostic group C); as time-dependent effect using one degree of freedom for its fit in model (DFTVC1); **Predicted FP model that incorporating: CAPRA-S score between 3 and 5 (CAPRA-S score group 2) and CAPRA-S score between 6 and 12 (CAPRA-S score group 3) with one degree of freedom for the restricted cubic spline function used for the baseline hazard rate (DF2).

**Figure 6.**
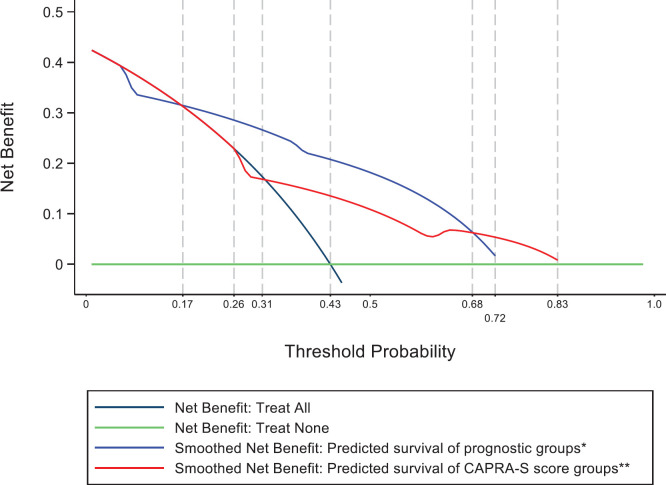
Decision curve analysis for FP model of MRD prognostic groups and FP model of CAPRA-S score groups on 347 men with and without biochemical failure treated by radical prostatectomy for prostate cancer followed for 10 years.

Figure 6 shows the results of the decision curve analysis for the FP model of MRD prognostic groups and FP model of CAPRA-S score groups for the range of probability threshold values observed between 0 and 1. In men treated by radical prostatectomy and followed for 10 years for a probability threshold of 0.17–0.68, the model based on the MRD prognostic groups was superior to the model based on CAPRA-S score groups. For a threshold probability smaller than 0.17, the CAPRA-S score model was similar to the strategy treat all. Similarly, for a threshold probability higher than 0.68, the CAPRA-S score model was similar to the MRD prognostic groups for predicting biochemical failure.

## Discussion

The CAPRA-S score for predicting future biochemical failure after radical prostatectomy has been externally validated and divides patients into low-, intermediate- and high-risk groups. The concordance index (C-index) between predicted biochemical failure-free survival (CAPRA-S) and observed biochemical failure-free survival (Kaplan–Meier) has been reported to be between 0.70 and 0.80 at 5 years and 0.66 and 0.80 at 10 years [8, 21–23]. The results showed a C-index of 0.69 and observed biochemical failure-free survival rates at 10 years similar to the published data [22, 23]. The CAPRA-S score is based on known prognostic risk factors found in the surgical piece and the pre-surgical serum PSA. However, not all cancer cells are equal, there is a heterogeneity in the phenotypic expression of tumour cells in the same patient. Some tumour cells are capable to disseminate early in prostate cancer, survive in the circulation and implant in distant tissues [24]. The morphological characteristics used to define the Gleason score do not identify these characteristics. Especially, in Gleason 7 patients (3 + 4 and 4 +3), there is a heterogeneity in clinical outcomes, and more recently, genomic testing has revealed differences that predict indolent Gleason 7 cancers from aggressive lethal ones [25, 26]. Within the same patient, there is a considerable variability in genomic alterations found in biopsy cores [27, 28]. The parameters used to determine the CAPRA-S score are fixed, and thus, the changes with time in the biological characteristics of disseminated tumour cells in the blood and/or bone marrow will not be reflected in the risk score.

That patients with negative surgical margins progress to biochemical failure, implying that there is local or distant dissemination of cancer cells that are not detected by conventional studies. This is termed as MRD, and we have previously reported that there at least two subtypes of MRD: first, those patients with CPCs detected in the circulation and second, those patients with only bone marrow micrometastasis. The presence of CPCs independent of whether bone marrow micrometastasis is present or not is associated with an increased risk of early failure, whereas those with only bone marrow micrometastasis are at risk of late failure [9]. The presence of CPCs, even in patients with pT2 disease, Gleason 6 and negative surgical margins is associated with early treatment failure. However, the frequency of such patients is significantly lower than patients with pT3a margins negative and pT3a margins positive, 20% versus 58% and 65%, respectively [29]. The significantly higher frequency of MRD negative disease in pT2 margin negative patients is one explication for the better prognosis. Interestingly, the frequency of patients with only bone marrow micrometastasis was similar in the three groups [29]. Positive surgical margins (PSM) have been associated with an increased risk of biochemical failure. Recently, a single PSM of >3 mm or multifocal PSM was associated with an increased risk of metastasis [30], whereas a single focus of PSM was not associated with biochemical failure [31]. Interestingly, the frequency of MRD subtypes detected was not significantly different between patients with pT3a PSM negative and positive prostate cancer although there were a limited number of pT3a-positive PSMs in the study [29].

What is important in the group of patients with only bone marrow micrometastasis (Group B in this study) is that, for the first 5 years, the biochemical failure-free survival is similar to those patients MRD negative. This is explained by the concept of dormancy. The interactions between the tumour cell and microenvironment, including the immune system, determine whether tumour cells proliferate or remain in a quiescent state. This quiescent state may last for years and is seen in the clinical as the time between primary treatment and failure in patients without evidence of metastatic disease. Changes in the tumour cells such as clonal progression [30] or changes in immune surveillance may lead to tumour activation. The CAPRA-S score was not significantly different between men MRD negative and those micrometastasis positive (Group B), and thus, unlike the MRD prognostic classification was unable to identify those men at risk of late failure. Similarly, in the CPC positive high-risk group, the CAPRA-S score classified over one-third of patients as low risk. The risk of biochemical failure in Group B patients changes with time, and thus, analysis using the Cox proportional hazard model is not applicable. We have previously reported that the use of CPC detection combined with the CAPRA-S score significantly improved the predictive ability of the prognostic model for early biochemical failure [10]. This new prognostic model improves further the predictive value, identifying men with the risk of late failure and who for the first 5 years appear to be in remission. The Harrell’s C index of the MRD prognostic test was superior to the CAPRA-S score in predicting biochemical failure-free survival.

The clinical usefulness of detecting the subtypes of MRD depends on its ability to bring benefits for patients by differentiating the following alternate therapies: early adjuvant or salvage therapy if CPCs are detected; if micro-metastasis is also present, the implication is that local radiotherapy will not be sufficient and hormonal therapy may be more beneficial. Patients with only micrometastasis detected long-term follow-up and hormonal therapy at biochemical failure, and finally, those patients negative for MRD may require less frequent follow-up. The decision curve analysis was used to determine the net benefit of a medical decision, which is the difference between the benefit and harms of treatment [31]. In this study, the MRD prognostic evaluation was superior to the CAPRA-S score. Thus, we consider that the MRD prognostic model gives clinically significant information to aid the decision on who may be eligible for adjuvant therapy, the type of therapy systemic or local, the timing for early or late failure and, conversely, those patients who may not need adjuvant therapy. The use of MRD detection permits the sequential follow-up of patients, detecting changes in the biological characteristics of tumour cells detected in blood and bone marrow and thus permitting changes in risk classification and/or treatment decisions.

The use of pre-operative PET-CT may also have a use in the possible therapeutic strategy for patients with intermediate–high-risk prostate cancer. The use of Gallium-68-prostate specific membrane antigen (^68^GA-PMSA) positron emission tomography/computed tomography predicted complete biochemical response from radical prostatectomy and lymph node dissection in these patients [32]. However, a recent review concluded that the sensitivity to detect lymph node metastasis pre-surgery was only moderate and should not replace lymph node dissection [33]. The use of ^64^copper-PMSA-617 PET/CT was superior to ^18^F-chiline PET/CT in restaging after biochemical failure [34]. In pre-surgery staging, this method showed a sensitivity of 87.5%, specificity of 100%, PPV of 100% and NPV of 93.7% of infiltrated lymph nodes and thus may be a useful adjuvant in pre-treatment staging [35]. There has been an increasing use of multiparametric magnetic resonance imaging (mpMRI) to stage prostate cancer; it is a non-invasive diagnostic tool with a high negative predictive value. The European Association of Urology guidelines recommend mpMRI when a patient is considered for active surveillance [36]. A recent meta-analysis of 28 studies reported that mpMRI is useful to better select the ideal candidates for active surveillance and to monitor them during follow-up. However, despite many advantages, there are still important limitations to detect all clinically significant prostate cancer, the need to better define mpMRI-radiological progression during the period of active surveillance and to rule out potentially aggressive disease [37]. As an imaging method it does not identify the biological properties of cancer cells nor the detection of micro-metastasis (0.2-2mm) which is beyond its resolution and thus mp-MRI is unable to detect MRD.

It has been suggested that obesity and testosterone levels are associated with adverse prostate cancer. A low serum testosterone levels (<300 ng/dL) has been significantly associated with upgrading, upstaging, unfavourable disease and positive surgical margins [38]. Similarly, obesity has been associated with adverse pathological features [39], and more specifically, the percentage of visceral adipose tissue is an independent predictor of adverse prognostic factors and low serum testosterone [40]. A small study suggested that the frequency of CPC detection increased with increasing body mass index in patients with an increased PSA [41]; however, there are no studies comparing obesity with MRD.

The study has several limitations; the detection of micrometastasis using bone marrow aspirations or biopsy has been documented although differing antibodies have been used to identify tumour cells, anti-cytokeratin, anti-PSA and anti-prostate specific membrane antigen (PSMA) for prostate cells. The use of reverse transcriptase-polymerase chain reaction for PSA and PSMA is reported to have ten times the sensitivity to detect tumour cells. However, detecting every cancer may not be important, and patients’ post-allogeneic bone marrow transplantation for leucaemia may have a very small number of leucaemic cells detected by RT-PCR in bone marrow samples but remain in remission for many years. Furthermore, these leucaemia cells may survive for prolonged periods before being eradicated by host defences [42]. As such ultrasensitive methods to detect tumour cells may overestimate clinically important minimal residual disease in patients with solid tumours.

We used anti-PSA which is specific for prostate and bone marrow biopsy touch-preps for three main reasons; first, the samples do not need to be decalcified or need an antigen recuperation process and as such epitopes are not destroyed; second, the diagnostic accuracy between touch-preps and biopsy samples is reported to be 84% and a positive correlation of 85% with the biopsy specimen [43]. Although thought to be an invasion procedure performed under sedation and local anaesthesia, the risk of adverse effects is minimum, less than 0.08% [44].

For the detection of CPCs, we used differential gel centrifugation and immunocytochemistry, acknowledging that the detection of CPCs or CTCs is method dependent. However, although the study had the disadvantage of being a single centre, it has the advantage of an immunocytologist who has the experience and training to perform the tests which have been internally validated as to pre-analytical, analytical and post-analytical variables as described in the methods section. Using the EpCAM (Epithelial Cell Adhesion Molecule)-based CellSearch® system, the frequency of patients positive for CPCs has been reported to be between 5% and 42% in patients with localised cancer [45, 46]. Comparing three different methods of CPC detection, the CellSearchÒ system detects CPCs in 14% of high-risk patients, the EPISPOT assay in 42%Ò of patients and 48% of patients using the CellCollectorÒ [47]. The differences and pitfalls of the different methods to detect CPCs have been reviewed [48]. The method that we used to detect CPCs is based on cell size and density but will not detect CPCs and micrometastasis, which do not express PSA. However, the use of standard immunocytochemistry has the advantage that it could be carried out in the routine laboratory of a general hospital without the need for high-cost technology or highly specialised personnel.

The results of the study need to be confirmed with a larger number of patients. However, the presented results show that risk classification based on morphological characteristics may not represent the biological characteristics of cancer in individual patients and thus not accurately predict the outcome.

## Conclusions

The CAPRA-S is an externally validated risk classification based on the pre-treatment PSA level and pathological findings in the surgical specimen. Three risk groups—low, intermediate and high—have been identified on which to base treatment decisions and have an acceptable predictive value. The MRD prognostic classification is based on the biological characteristics of the tumour cell–microenvironment interaction, to give three groups: MRD negative, only bone marrow micro-metastasis and CPC positive prostate cancer. Differing from the CAPRA-S score classification, the risk of treatment failure changes with time, differentiating between early and late treatment failures. The CAPRA-S does not differentiate between MRD negative and micrometastasis only prostate cancer patients, and the high-risk CPC-positive patients may be classified as low-risk CAPRA-S. The study results warrant a further larger-scale confirmation.

## Conflicts of interest

Dr Murray has received consultancy fees from Viatar CTC solutions, Boston, USA.

## Funding

The study was funded by a Hospital de Carabineros de Chile research grant. The funding source had no involvement in the study design, collection, analysis and interpretation of the data, in the writing of the report and in the decision to submit the article for publication.

## Authors’ contributions

Concept: NPM; design: NPM; supervision: NPM, ER and CF; resources: NPM; materials: NPM, SO, CF, AS and ER; data collection and/or processing: CF, AS, ER, EG and SO; analysis and/or interpretation: NPM, SO, AS, CF, ER, EG and SO; literature search: SO and EG; writing manuscript: NPM and EG; critical review: CF, AS, ER and SO; final approval: all.

## Figures and Tables

**Table 1.** Clinicopathological features of the prognostic groups on 347 men with and without biochemical failure treated by radical prostatectomy for prostate cancer followed for 10 years.

Characteristic	Group A Absence CPCs Absence mM *n* = 155	Group B Absence CPCs Presence mM *n* = 56	Group C Presence CPCs Presence/Absence mM *N* = 136	*p*-value two-tail
Age at diagnosis mean± DS	64.82; 7.85	66.05; 10.50	66.13; 8.76	0.232 ^a^
PSA at diagnosis median; IQR	6.02; 1.61	5.69; 2.36	6.72; 4.94	<0.01 ^b^
Gleason score median; IQR ≤6 7 ≥8	5; 2 135 15 5	6; 1 46 8 2	7; 2 64 40 32	<0.01^b^
Pathological stage median; IQR	2;1	2;0	2;1	<0.01^c^
Surgical margins Positive n (%)	7 (4.2)	6 (10.71)	46 (33.82)	<0.01^d^
extra-capsular extension present n (%)	31 (20.00)	15 (26.79)	93 (68.38)	<0.01^d^
Seminal vesicle Infiltration present n (%)	0 (0)	0 (0)	10 (7.35)	<0.01^e^
Lymph node infiltration present *n* (%)	0 (0)	0 (0)	4 (3.10)	0.037^e^
CAPRA-S median; IQR 0–2 3–5 ≥6	0:1 134 20 1	1:2 48 6 2	3:5 51 44 41	<0.01^b^ <0.001^e^

DS = standard deviation; IQR = interquartile range; mM = micrometastasis; CPC = circulating prostate cell.

a Kruskal–Wallis test;

b Kruskal–Wallis test (significant difference between groups: A versus C and B versus C);

c Kruskal–Wallis test (significant difference between groups: A versus B, A versus C and B versus C);

d Marascuillo procedure (significant difference between groups: A versus C and B versus C);

e Fishers’ Exact tests.

**Table 2.** Observed biochemical failure-free survival (Kaplan–Meier) versus predicted biochemical failure-free survival for FP model by prognostic groups and FP model by CAPRA-S score groups on 347 men with and without biochemical failure treated by radical prostatectomy for prostate cancer followed for 10 years.

Type		Observed^a^		Predicted	
		Survival % (95% CI)	RMST years (95%CI)	Survival % (95% CI)	RMST years (95%CI)
Prognostic group	Group A CPCs and mM negative *n* =155	95.43 (90.04–97.93)	9.78 (9.61–9.95)	92.23 ^b^ (83.33–96.48)	9.84 ^b^ (9.71–9.97)
	Group B CPCs negative and mM positive *n* = 56	56.73 (38.07–71.69)	9.35 (9.03–9.67)	62.43 ^b^ (45.82–75.26)	9.16 ^b^ (8.76–9.55)
	Group C CPCs positive *n* = 136	26.82 (19.41–34.77)	6.13 (5.59–6.68)	27.33 ^b^ (20.00–35.15)	6.11 ^b^ (5.57–6.64)
	All subjects n = 347	57.03 (50.41–63.11)	8.19 (7.89–8.49)	61.99 ^b^ (58.85–65.12)	8.27 ^b^ (8.08–8.45)
CAPRA-S score groups	Group 1 CAPRA-S score between 0 and 2 *n* = 234	68.46 (60.02–75.48)	9.05 (8.78–9.32)	72.65 ^c^ (65.58–78.50)	8.90 ^c^ (8.62–9.19)
	Group 2 CAPRA-S Score between 3 and 5 *n* = 70	47.39 (34.51–59.21)	6.93 (6.16–7.70)	37.03 ^c^ (24.61–49.46)	7.17 ^c^ (6.46–7.88)
	Group 3 CAPRA-S score between 6 to 12 *n* = 43	15.91 (6.10–29.87)	5.66 (4.71–6.61)	16.04 ^c^ (7.41–27.60)	5.74 ^c^ (4.87–6.61)
	All subjects *n* = 347	57.03 (50.41–63.11)	8.19 (7.89–8.49)	58.45 ^c^ (56.20–60.69)	8.16 ^c^ (8.04–8.28)

FP = flexible parametric; CPCs = secondary circulating prostate cells; mM = micrometastasis; %: percentage;

a Observed used the Kaplan–Meier survival model;

b Predicted FP model that incorporating: Mm positive and CPCs negative (prognostic group B), CPCs positive (prognostic group C) with two degrees of freedom for the restricted cubic spline function used for the baseline hazard rate (DF2) and also, consider the CPCs positive (prognostic group C); as time-dependent effect using one degree of freedom for its fit in model (DFTVC1);

c Predicted FP model that incorporating: CAPRA-S score between 3 and 5 (CAPRA-S score group 2), CAPRA-S score between 6 and 12 (CAPRA-S score group 3) with one degree of freedom for the restricted cubic spline function used for the baseline hazard rate (DF2).
